# Intracardiac Repair in Late Adolescent and Adult Tetralogy of Fallot — Early and Midterm Results from a Tertiary Care Centre

**DOI:** 10.21470/1678-9741-2020-0528

**Published:** 2022

**Authors:** Rahul Bhushan, Vaibhav Chugh, Manpal Loona, Javed Bandey, Narender Singh Jhajhria, Vijay Grover, Vijay Kumar Gupta

**Affiliations:** 1 Department of Cardiovascular and Thoracic Surgery, ABVIMS and Dr Ram Manohar Lohia Hospital, New Delhi, India.

**Keywords:** Adult, Tetralogy of Fallot, Heart Septal Defects, Ventricular, Pulmonary Artery, Postoperative Period, Treatment Outcome, Patients Rights

## Abstract

**Introduction:**

In developing countries like India, it is common for late presentation of Tetralogy of Fallot (TOF) patients to a hospital as compared to that of developed countries. The objective of this study is to analyze the surgical outcome of TOF patients with age > 15 years.

**Methods:**

This is a retrospective descriptive study of the surgical outcomes of 45 adult patients undergoing correction for TOF. Epidemiology, symptomology, and preoperative evaluation were performed.

**Results:**

Most of the patients were male (33 [73%]). The median age was 21 years. A total of 42 (93.33%) patients had subaortic ventricular septal defect (VSD), while three (6.6%) patients presented with doubly committed VSD. The most common type of right ventricular outflow tract (RVOT) obstruction was combined infundibular and valvular types, accounting for 34 cases (75.5%). Six patients had infundibular RVOT obstruction, while three patients (6.6%) had predominantly valvular pulmonary stenosis. We performed trans-right atrial repair in 33 patients. Right atrium-pulmonary artery approach was used in five patients (11.1%). The most common postoperative complication was right bundle branch block, seen in 14 patients, with a mortality rate of 2% in the early postoperative period. We achieved excellent early and midterm survival results and significant improvement in functions and disease-free quality of life.

**Conclusion:**

Intracardiac repair in adult TOF can be performed with low mortality, less residual RVOT obstruction, and need for revision of RVOT far less frequent by using the Jhajhria Infundibular Resection Adequacy Assessment technique (JIRAAT) to assess for adequacy of infundibular resection.

**Table:** 

Abbreviations, acronyms & symbols			
ASD	= Atrial septal defect	PFO	= Patent foramen ovale
CATH	= Catheterization	PI	= Pulmonary insufficiency
CPB	= Cardiopulmonary bypass	PR	= Pulmonary regurgitation
ECHO	= Echocardiography	RA	= Right atrial
ICU	= Intensive care unit	RBBB	= Right bundle branch block
JET	= Junctional ectopic tachycardia	RV	= Right ventricular
JIRAAT	= Jhajhria Infundibular Resection Adequacy Assessment Technique	RVOT	= Right ventricular outflow tract
LAD	= Left anterior descending coronary artery	TOF	= Tetralogy of Fallot
MPA	= Main pulmonary artery	TR	= Tricuspid regurgitation
PA	= Pulmonary artery	VSD	= Ventricular septal defect

## INTRODUCTION

Tetralogy of Fallot (TOF) is the most common cyanotic congenital heart disease, accounting for 10% of congenital heart defect patients and occurring in one in 3,600 births^[1]^. The 10-year survival rate in untreated patients is 24% only^[2]^.

The basic anatomical defects stem from the anterior and cephalad deviation of the infundibular septum. Less severe right ventricular outflow tract (RVOT) obstruction or associated major aortopulmonary collaterals may lead to delayed appearance of symptoms, and thus late presentation of patient for evaluation. Only a minority of patients with unrepaired TOF reach adulthood without symptoms or complications such as haemoptysis, intracerebral abscesses, etc. In developing countries like India, it is common the late presentation of TOF patients to a hospital as compared to that of developed countries, which may be due to financial constraints, neglecting symptoms in poor socioeconomic strata patients, delayed referral to tertiary care centre, and limited availability of paediatric cardiac surgery centres.

Nevertheless, the natural history of the chronic hypoxia that characterizes TOF is responsible for a variety of complications such as cerebral complications, myocardial dysfunction, and propensity to ventricular arrhythmias^[3^-^5]^.

We performed a retrospective analysis of total correction for TOF in adults with age > 15 years to analyze surgical outcome in this specific subset of TOF.

## METHODS

A retrospective descriptive study was designed to study the in-hospital outcome of all the adult patients who had undergone intracardiac repair for TOF from January 2015 to January 2020 at Atal Bihari Vajpayee Institute of Medical Sciences (or ABVIMS) and associated Dr Ram Manohar Lohia Hospital (New Delhi, India), a tertiary care centre.

A total of 362 cases of complete repair for TOF were performed during this interval in our centre, out of which 45 were adult TOF patients, *i.e.*, age > 15 years (12.43%). All symptomatic patients with defined age criteria diagnosed with TOF and having favorable anatomy for complete repair for TOF were included in our study. TOF/pulmonary atresia and TOF/pulmonary atresia/major aortopulmonary collateral arteries requiring unifocalization or those requiring palliative shunt for severe cyanosis at presentation to us were excluded from the study. Epidemiology, symptomatology, and preoperative evaluation using routine echocardiography (ECHO) and cardiac catheterization (CATH) were done in all patients. Routine follow-up with weekly follow-up for six weeks, monthly follow-up for six months, following half yearly follow-up were done.

### Surgical Technique

A uniform operative technique was used with bicaval cannulation for cardiopulmonary bypass (CPB) and moderate systemic hypothermia. Any patent shunts were taken down. The ductus arteriosus was dissected and ligated if present. Main pulmonary artery (MPA) separated from aorta and both its branches were fully mobilised, which aids in pulmonary valve inspection through the right atrium. Myocardial protection was achieved with Del Nido cold blood cardioplegia delivered through aortic root, supplemented with ice slush on the myocardium, and repeated if required at 100 minutes. Following the cardioplegic arrest, right atrium was opened by an incision starting from the base of the right atrial (RA) appendage and extending to the medial aspect of the inferior vena cava. The left side of the heart was vented through patent foramen ovale (PFO) or atrial septal defect (ASD). If none were present, a stab incision was made for venting at fossa ovalis. Patients were cooled to 28-30 °C. Excessive return to left atrium was managed with increasing the negative suction on vent and, if necessary, by decreasing the arterial flow with further hypothermia to 26 °C.

Two everting 5-0 Prolene stay sutures were placed on the right atrium just anterior to tricuspid valve and hanged over the left sternal blade of retractor. The ventricular septal defect (VSD) suture was taken through base of septal tricuspid leaflet and retracting it to the RA side for visualization. The anterior tricuspid leaflet was also gently retracted using a right-angled retractor with half inch blade. Via a right atriotomy and working through the tricuspid valve, the parietal extensions of the infundibular septum were divided parallel to the aortic annulus up to the level of the pulmonary valve. The dissection was completed by excision of the obstructing parietal bands, anterior infundibular trabeculations, and the septal bands. In all cases, the pulmonary valve was inspected by holding the leaflets, and any tethering, if seen, was released; a commissurotomy was done when needed to relieve any stenosis. Additional 5-0 Prolene stay sutures were placed on pulmonary annulus at 12 o’clock in difficult cases for better visualisation for pulmonary valvotomy and detethering the leaflets. Hegar dilators were then used to assess the size of the pulmonary annulus.

Ventriculotomy or transannular patch was avoided by liberally coring the RVOT and accepting a pulmonary annulus of a Hegar probe that was two sizes smaller than the mean diameter listed on the Rowlatt chart. Both branched pulmonary arteries (PA) were assessed with Hegar dilator (half size) as per Rowlatt chart.

Adequacy of RVOT resection was assessed by Jhajhria Infundibular Resection Adequacy Assessment Technique (JIRAAT). Keeping the index finger inside the RVOT and the thumb outside, we could assess the residual anterior bands and the thickness of RVOT to prevent over resection or under resection. This technique supplemented visual interpretation of the RVOT through the tricuspid valve.

The VSD was then closed using interrupted Prolene (5-0) sutures for the patients weighing < 20 kg and with Prolene (4-0) suture for those weighing > 20 kg. The sutures were pledgeted by Dacron of approximately 3 mm*2 mm pledgets. Tailored Dacron patch was used to close the VSD from the 3 o’clock position, in clockwise direction, with extra care in posteroinferior segment to protect conduction tissue and lifting tricuspid leaflet to prevent suture entrapment. The tricuspid valve was assessed for competence by saline insufflations and mild central tricuspid regurgitation (TR) was accepted. Three patients had moderate tricuspid insufficiency on saline insufflation and required tricuspid annuloplasty at the antero-septal commissure. A 4-5 mm PFO was left open at the expense of mild cyanosis since it allowed for complete repair without using a transannular patch.

Annular diameter adequacy was assessed with Hegar dilators. Transannular patch was avoided if Z-score for age was <-2. When annulus did not admit Hegar up to Z value-2, then a small, limited transannular patch was placed using autologous non fixed pericardium. Any localized stenosis of MPA/branched PA was augmented with autologous pericardial patch.

According to our operative protocol, RVOT resection, VSD closure, pulmonary annulus assessment followed by pulmonary valvotomy, if needed, and assessment of tricuspid valve function were accomplished in most of patients via a single incision in the right atrium. The RVOT pressure and gradient across it were assessed on the operating table only in cases where there was difficulty in weaning from CPB or in view of poor hemodynamics. All postoperative patients were evaluated by ECHO prior to discharge.

### Follow-up

Routine follow-up with weekly follow-up for six weeks, monthly follow-up for six months, following half yearly follow-up were done. After that, patients were followed annually. The patient review was stratified based on age of presentation, presenting symptoms, ECHO and CATH findings, and surgical outcomes till August 2020.

## RESULTS

A total of 45 patients were included in the study from January 2015 to January 2020. Males comprised the majority of patients (33 [73%]); 12 (27%) were female. The median patient age was 21 years (range 15-44 years). Two patients (4%) had a modified Blalock-Taussig shunt placed in infancy at another centre.

The preoperative characteristics of the patients included in the study is shown in Table 1.

**Table 1:** Preoperative characteristics of the patients included in study.

Characteristic	Total number of patients	Percentage
Previous palliation	2	4%
Major aortopulmonary collateral arteries	19	42%
Atrial septal defect	15	33.3%
Left superior vena cava	3	6%
Patent ductus arteriosus	7	15.5%
History of cerebral abscess	6	13.3%
LAD crossing RVOT	1	2%

LAD=left anterior descending coronary artery; RVOT=right ventricular outflow tract

Nineteen patients (42.2%) had aortopulmonary collaterals significant enough to warrant preoperative coiling. ASD was present in 15 patients who underwent surgery (33.33%). Seven patients had placement of a transannular patch; in those with a pulmonary valve annulus Z-score <-2, the PFO was left open. Patent ductus arteriosus was noted in 15.5% of patients and it was ligated. A total of 42 (93.33%) patients had a subaortic VSD, while three (6.6%) patients presented with a doubly committed VSD.

The most common type of RVOT obstruction was combined infundibular and valvar type accounting for 34 cases (75.5%). In six patients (13.3%), RVOT obstruction was pure infundibular, while three patients (6.6%) had pure valvular obstruction. Two patients had localised hypoplasia of MPA or ostial stenosis at one or both of the branch PAs for which pericardial patch augmentation was done.

One patient had the left anterior descending coronary artery crossing RVOT and trans-RA total correction was done successfully.

As our institutionally preferred approach, we performed a trans-RA repair in 33 patients (73.3%), while a right atrium-PA approach was used in five patients (11.1%). A limited right ventriculotomy was done in the few patients who had placement of a transannular patch. Also, three patients that had significant residual gradients across the area of PA patch augmentation required revision prior to chest closure. One required revision of the MPA patch, one of the MPA-left PA patch, and one of the MPA-right PA patch.

Table 2 shows the surgical data and Table 3 describes the postoperative complications encountered. The most common complication in the postoperative period was right bundle branch block (RBBB), seen in 14 patients (31.1%). Two patients required reoperation for bleeding, and we had one early postoperative death (2%), attributed to right ventricular (RV) dysfunction with junctional ectopic tachycardia (JET). In our initial experience, there were six patients who required reinstitution of CPB for inadequate RVOT resection; based on *P*^RV^ and *P*^LV^ assessment (*P*^RV^:*P*^LV^ > 0.8 )and hemodynamic considerations.

**Table 2:** Surgical data.

CPB time (min)	100±3
Cross-clamping time (min)	77±1
Intubation time (hours)	11±2
ICU stay (hours)	42±1
Hospital stay (days)	4±1

CPB=cardiopulmonary bypass; ICU=intensive care unit

**Fig. 1:**
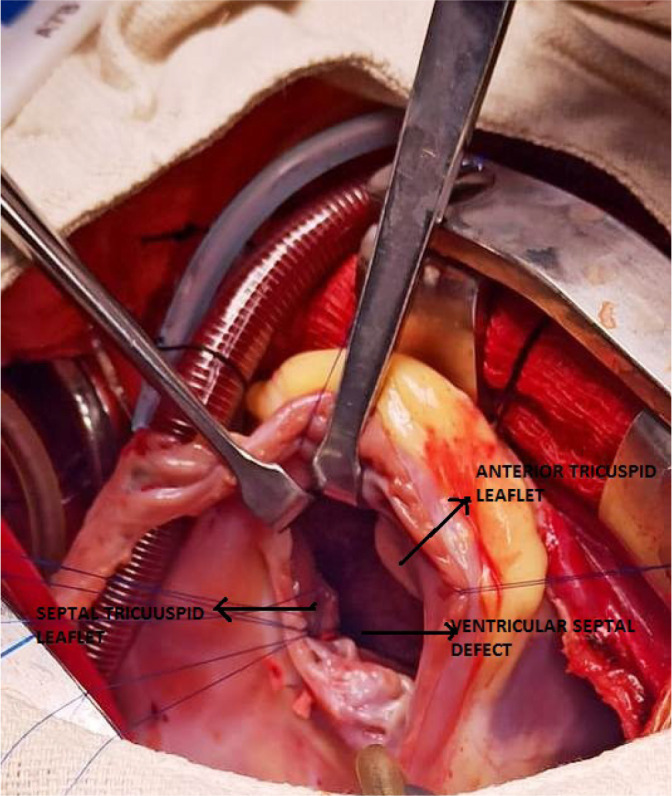
Intraoperative picture.

An intraoperative picture can be seen in Figure 1.

**Table 3:** Postoperative complications.

Exploration for bleeding	2
RBBB	14
Wound infection	0
Neurological complications	0
Cardiac arrhythmia (JET)	1
Complete heart block	0
Early deaths	1

JET=junctional ectopic tachycardia; RBBB=right bundle branch block

Since instituting the JIRAAT for evaluation of the adequacy of RVOT resection, no patients have required intervention for residual RVOT obstruction.

The average hospital length of stay in our study was five days (± 2 days).

A routine postoperative ECHO was performed in all patients prior to discharge and a satisfactory result of VSD closure with adequate RV coring was uniformly achieved in all patients.

### Follow-up

In this study, there was no late postoperative mortality at follow-up.

Mild pulmonary regurgitation (PR) was seen in five patients (11.1%), moderate in two (4%), and no patients demonstrated severe PR at follow-up.

Mean RVOT gradient on follow-up was found to be 12 [±5] mmHg.

Mild TR was noted in one patient in the follow-up period, while TR did not worsen in any other patient in our series. None of our patients developed significant RV functional impairment in the follow-up period.

## DISCUSSION

A subset of patients with TOF and minimal RVOT obstruction present in early adulthood^[1^,^2^,^6]^.

Of the 362 patients that had complete repair of TOF in our centre over the study period, 45 (12.43%) were adults. These patients face effects of prolonged cyanosis resulting into RV hypertrophy, polycythemia, coagulation defects, and the development of extensive bronchial collaterals^[3]^.

Complete clinical evaluation gives insight for the planning of intervention as well. Cardiac CATH and angiography are important diagnostic modalities for characterizing the cardiac and vascular anatomy and physiology in patients with TOF. This is especially true when planning a complete repair for patients with long-standing cyanosis and the development of extensive bronchial collaterals^[5^,^7]^. At our centre, we have made it an institutional policy of performing routine CATH study prior to operative intervention in adult TOF. In our study, 19 patients (42%) had significant aortopulmonary collaterals on evaluation necessitating coiling by cardiologist preoperatively.

We achieved excellent early and midterm survival results and significant improvement in functions and disease-free quality of life. We had 2% mortality rate in early postoperative period compared to standard 3-4% mortality in other study groups^[4^-^11]^.

Significant RV hypertrophy due to chronicity of disease necessitated adequate myocardial protection and we used routinely Del Nido cardioplegia with timely repeat whenever needed along with cold hypothermia^[9^,^10]^.

Our routine and preferred approach via a transatrial correction of the TOF is associated with excellent early and midterm results.

Our results confirm that this approach is safe (low mortality), and it also contributes to the preservation of satisfactory RV function by preserving the pulmonary valve and avoiding ventriculotomy^[10]^. Mean RVOT gradient was 12 in postoperative ECHO which was comparable to other studies^[4^,^5^,^6^,^12]^.

Moderate PR was seen in two patients, while none had severe PR. Serial ECHO follow-up was done in these patients to monitor RV function and reintervention was not indicated in either of patients based on symptomology or progression of RV dysfunction in serial follow-up. Mild TR was seen in one postoperative patient, while there was no progression of TR in routine postoperative follow-up in the remaining patients. Strict fluid restriction and diuretics were given in follow-up. Trans-RA approach also resulted in lesser incidences of rhythm disturbances. One patient went into JET in postoperative period, while 14 patients had RBBB in postoperative period.

Ventriculotomy was needed only on indication of pulmonary valve repair and transannular patch correction when needed. VSD should be closed rigorously with interrupted reinforced sutures due to decreased tolerance of any residual VSD in TOF patients^[13]^. Our transatrial approach for VSD closure achieved 100% successful repair. We routinely used antifibrinolysis drugs to combat higher bleeding tendency^[10]^.

Adequacy of RVOT resection was established by JIRAAT, devised by our own institution, which resulted into satisfactory results and improved postoperative outcome^[14]^.

Transannular patch placement was necessary in seven (16.2%) patients, a reduced number compared to those reported by other groups^[14^-^16]^. Postoperative pulmonary insufficiency (PI) is a common and anticipated sequela of this approach. Of the seven patients who had transannular patch placement, five were left with mild PI, and two with moderate PI. We did not have any patients that demonstrated severe PI. We had one early death resulting from severe RV dilatation and dysfunction and associated JET. This patient belonged to the transannular patch group, had a loss of atrioventricular synchrony with tachycardia, and was refractory to Amiodarone therapy. Most of the evidence in the literature pointed out the benefits of timely repair of any significant pulmonary or tricuspid valve dysfunction^[17^-^18]^.

## CONCLUSION

Using JIRAAT for adequacy of infundibular resection, complete repair of TOF can be performed successfully with minimal residual outflow tract obstruction and low mortality.

**Table:** 

Authors' roles & responsibilities	
RB	Substantial contributions to the conception or design of the work; or the acquisition, analysis, or interpretation of data for the work
VC	Drafting the work or revising it critically for important intellectual content
ML	Drafting the work or revising it critically for important intellectual content
JB	Drafting the work or revising it critically for important intellectual content
NSJ	Substantial contributions to the conception or design of the work; or the acquisition, analysis, or interpretation of data for the work; Final approval of the version to be published
VG	Final approval of the version to be published
VKG	Final approval of the version to be published
